# Supplementary figures and images for: Markov state modelling reveals heterogeneous drug-inhibition mechanism of Calmodulin
Source: PLoS Comput Biol. 2022 Oct 7;18(10):e1010583. doi: 10.1371/journal.pcbi.1010583 (PMC9581412; doi:10.1371/journal.pcbi.1010583)

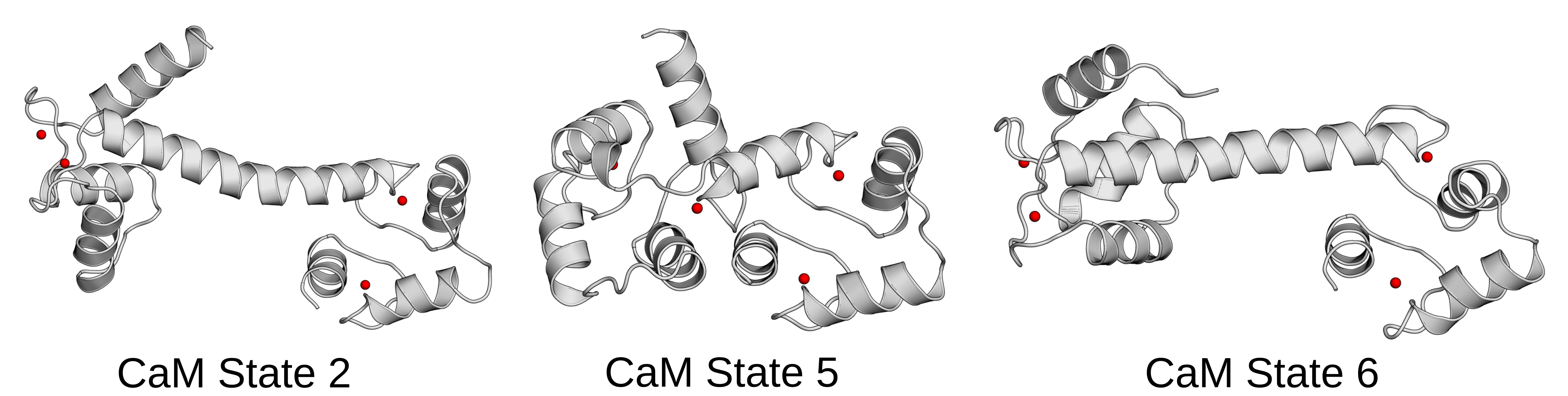

Supplement: S1 Fig — The bound Ca2+ ions included in the simulations are shown as red spheres. (TIFF) [file pcbi.1010583.s001.tiff]

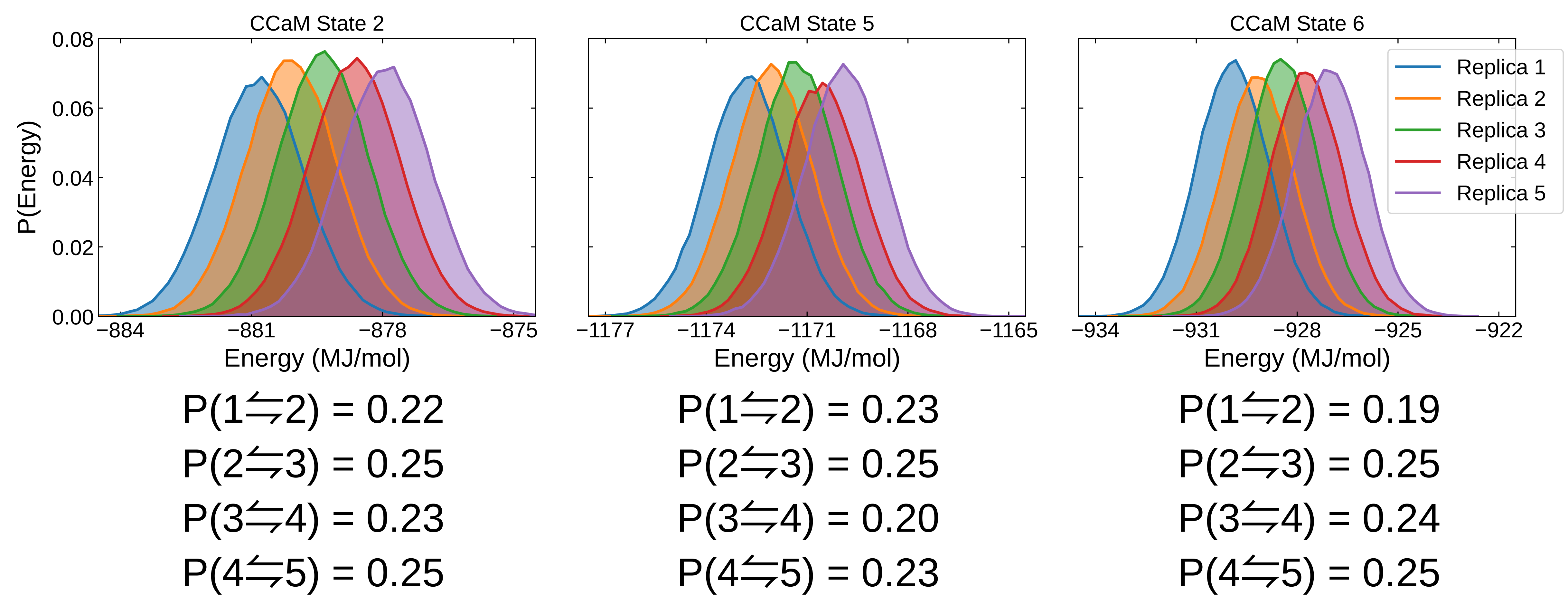

Supplement: S2 Fig — (TIFF) [file pcbi.1010583.s002.tiff]

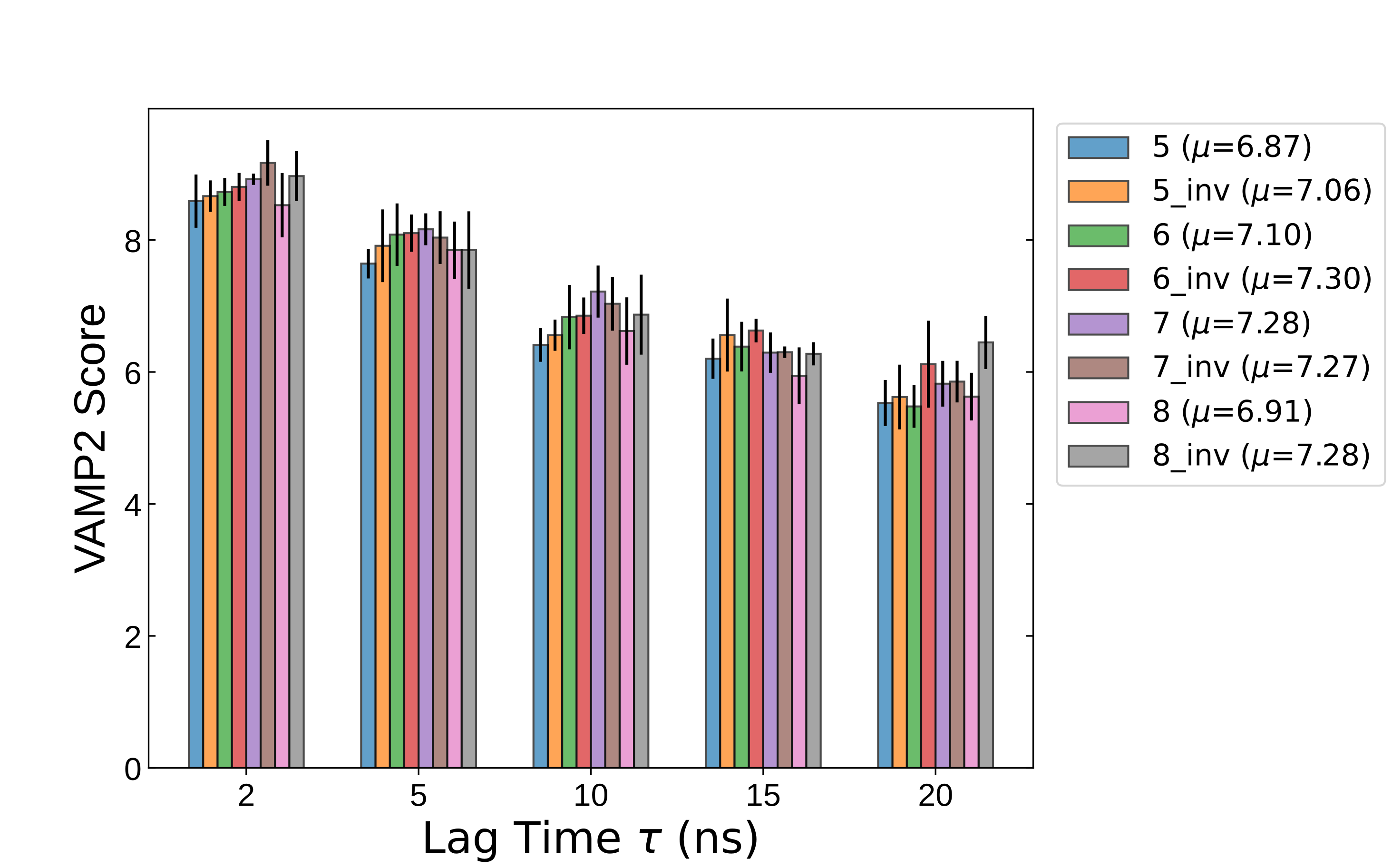

Supplement: S3 Fig — The mean values from five-fold cross-validation are plotted as bars and error bars represent the standard deviations. The mean value across the lag times calculated for each feature transformation is mentioned within the legend. (TIFF) [file pcbi.1010583.s003.tiff]

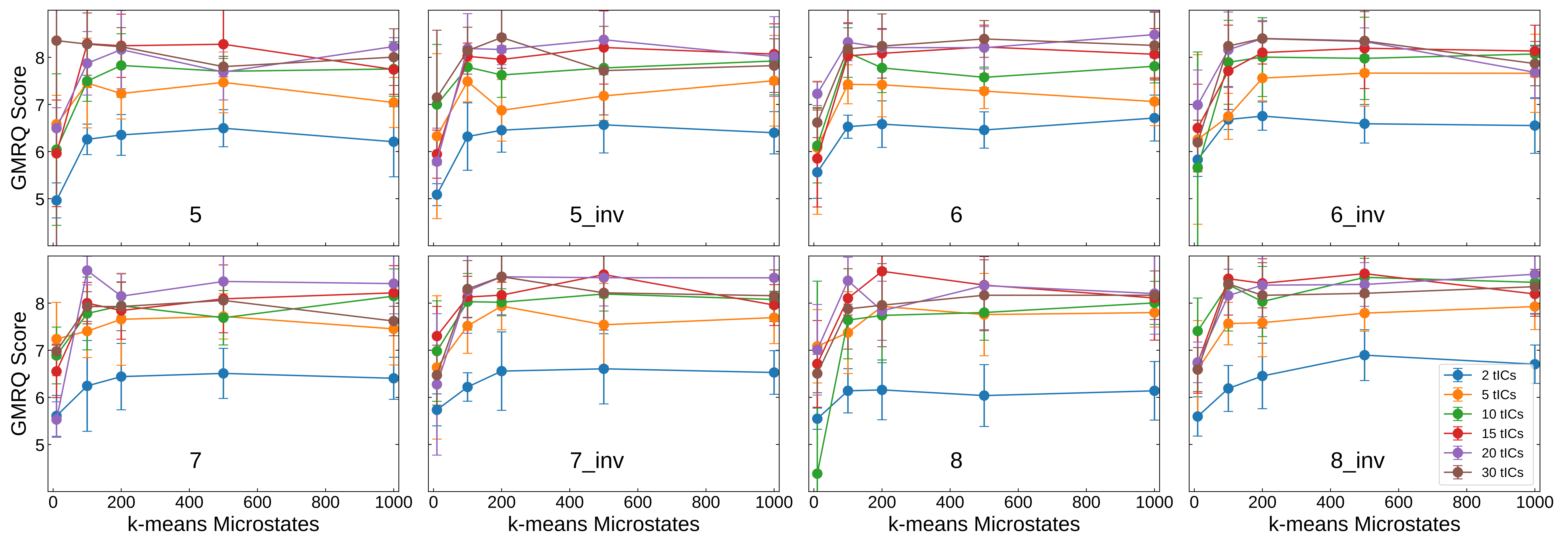

Supplement: S4 Fig — The mean values from five-fold cross-validation are plotted as dots and standard deviations are plotted as error bars. (TIFF) [file pcbi.1010583.s004.tiff]

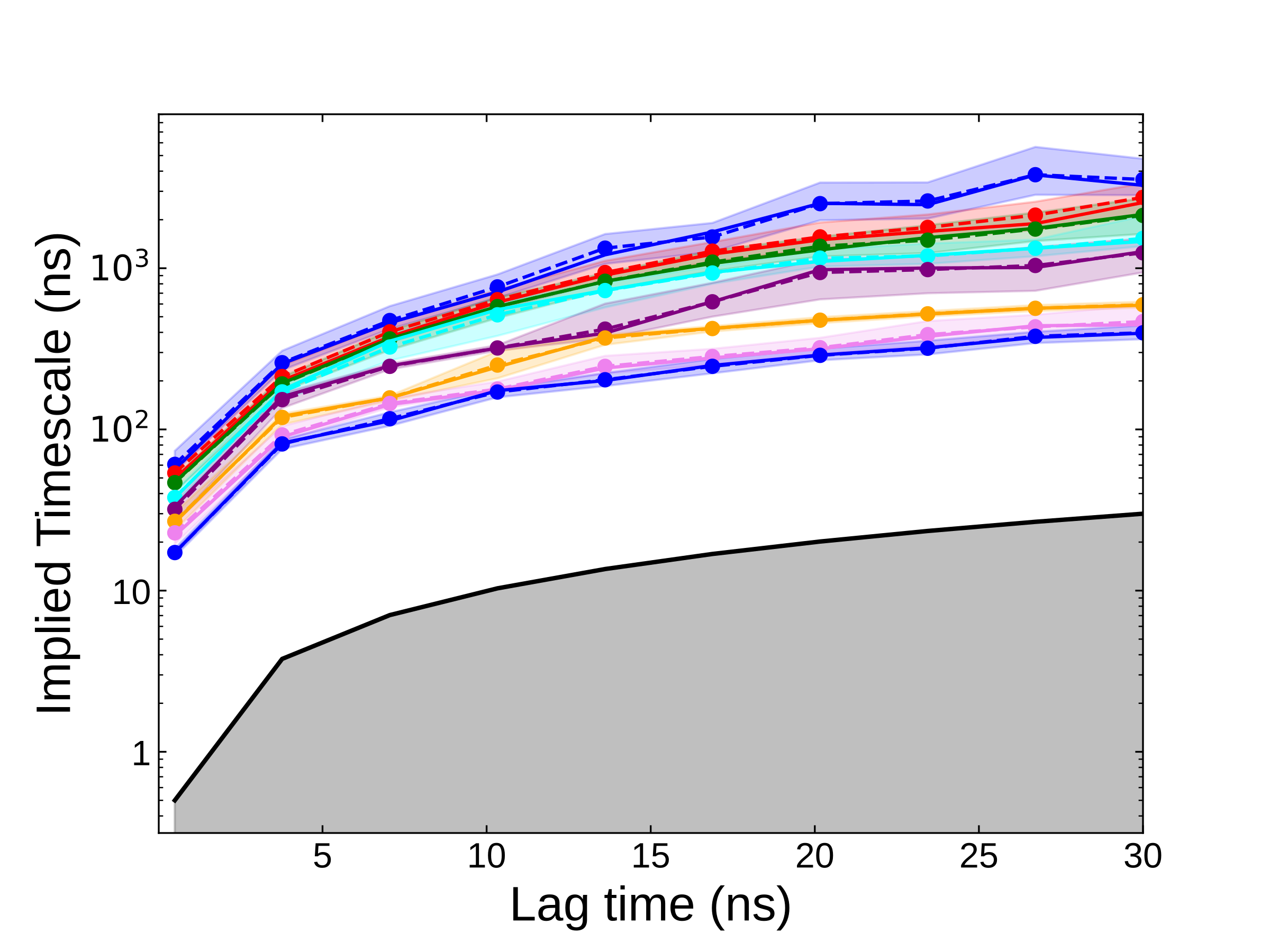

Supplement: S5 Fig — The 95% confidence intervals of the eigenvalues are shown as shaded regions. The black solid curve delimits a shaded region where the implied timescales are shorter than the lagtime. (TIFF) [file pcbi.1010583.s005.tiff]

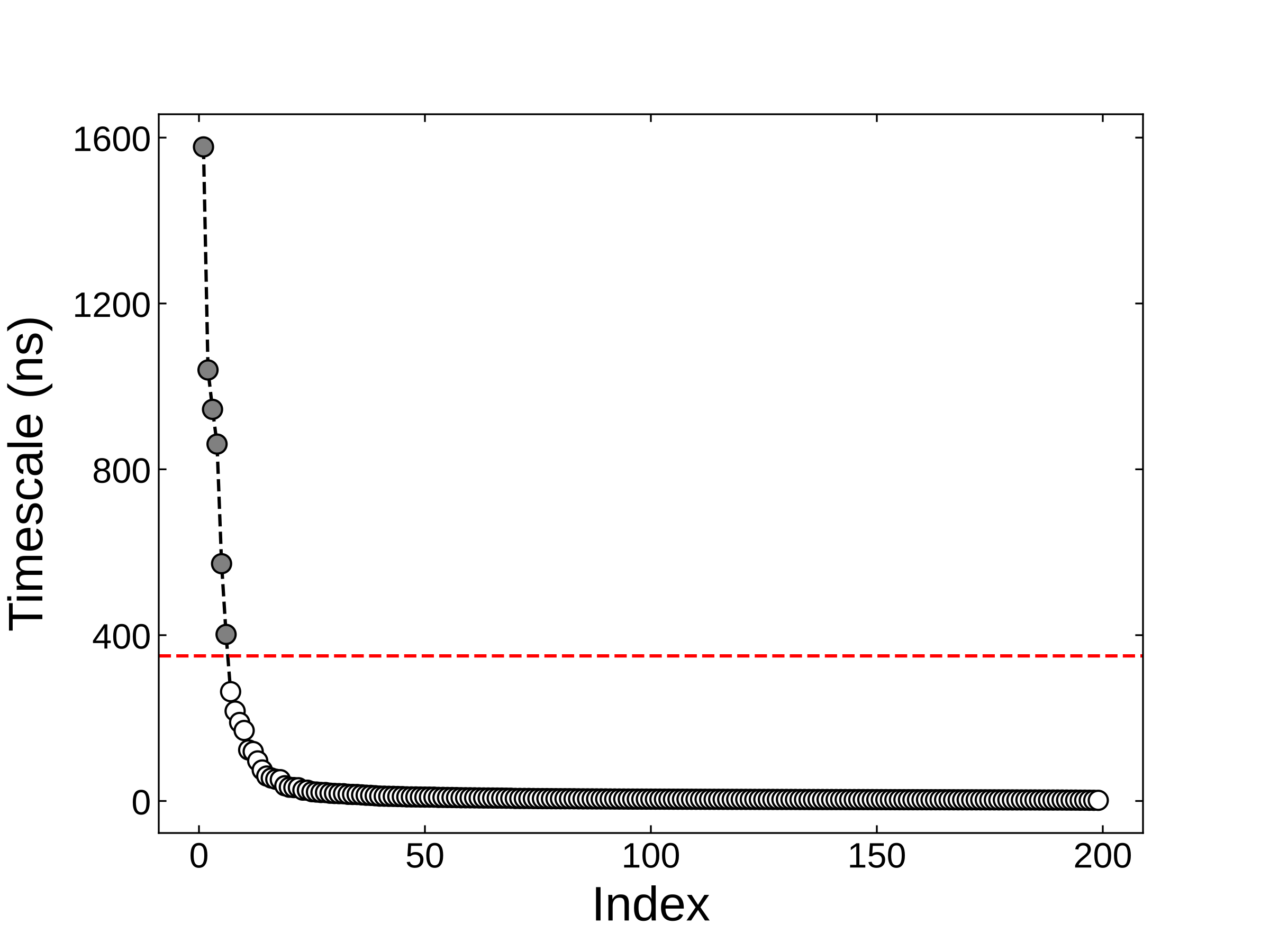

Supplement: S6 Fig — The cutoff between the sixth and seventh relaxation timescales selected in this work is illustrated as red dotted line. (TIFF) [file pcbi.1010583.s006.tiff]

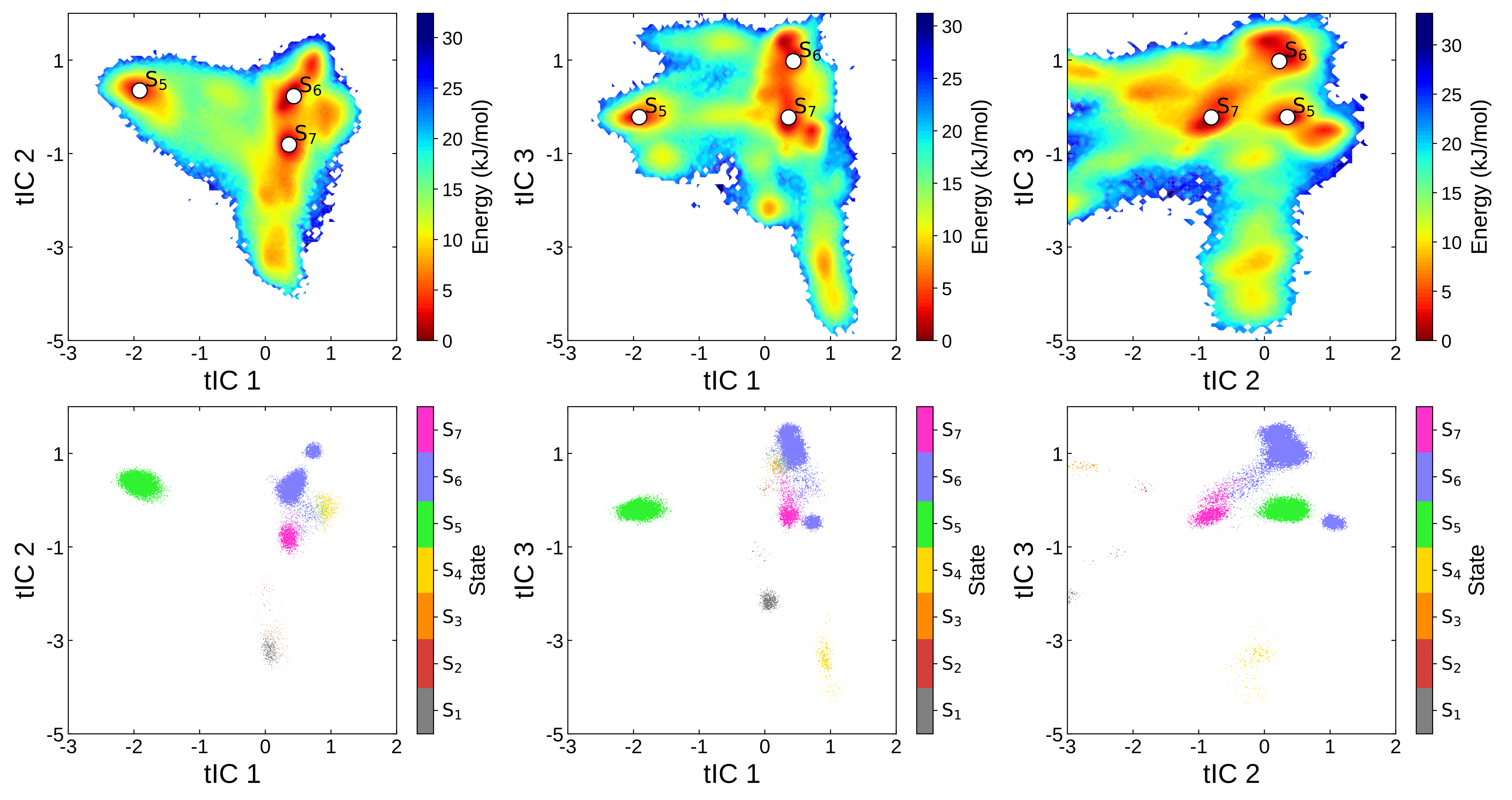

Supplement: S7 Fig — Bottom: S1-S7 macrostate assignments along the three tICs obtained from PCCA++ clustering. Each trajectory frame is represented as a dot within the scatter plot. (TIFF) [file pcbi.1010583.s007.tiff]

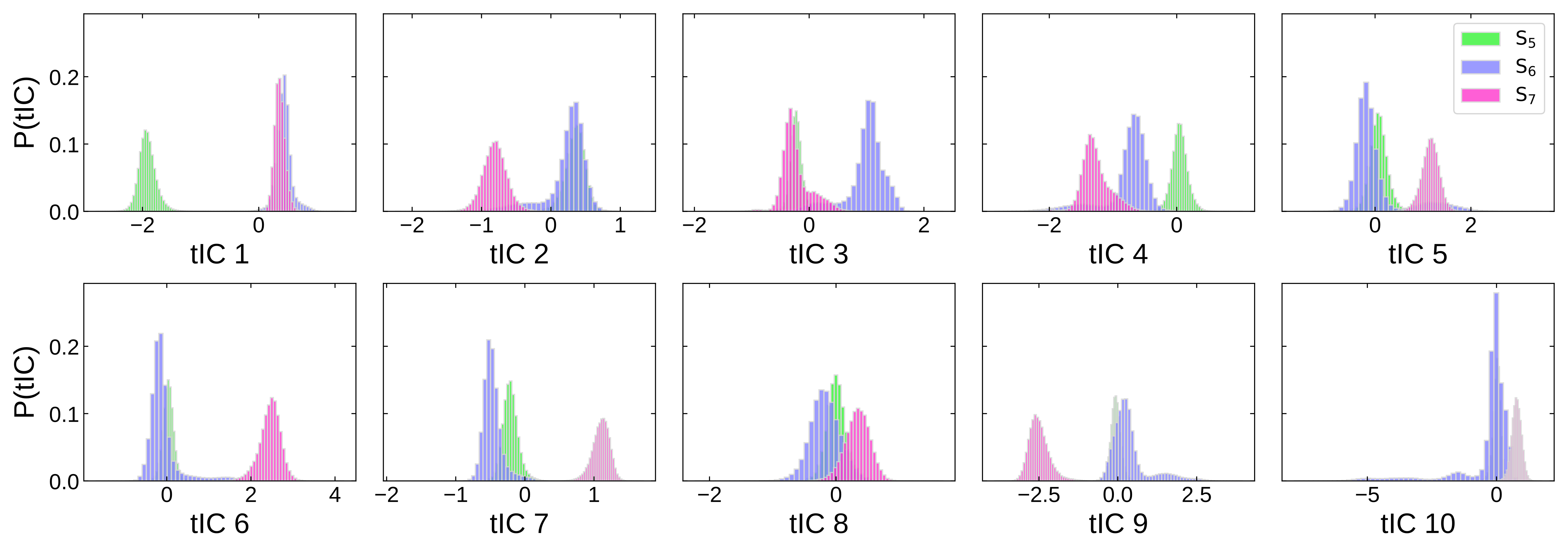

Supplement: S8 Fig — (TIFF) [file pcbi.1010583.s008.tiff]

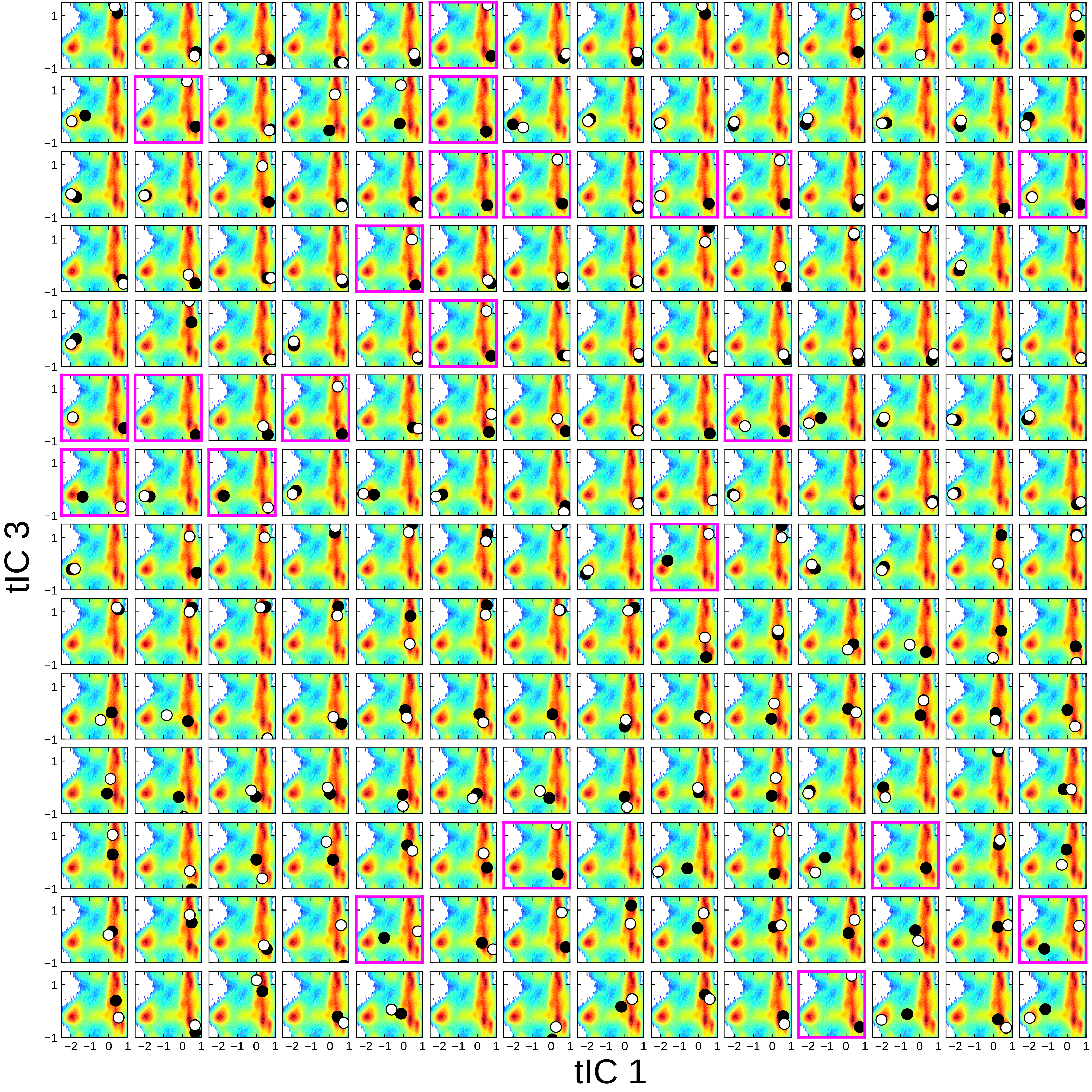

Supplement: S9 Fig — Individual trajectories are represented as subplots with the initial and final configuration shown as black and white dots respectively. Trajectories with transitions between the basins are highlighted with a magenta outline. (TIFF) [file pcbi.1010583.s009.tiff]

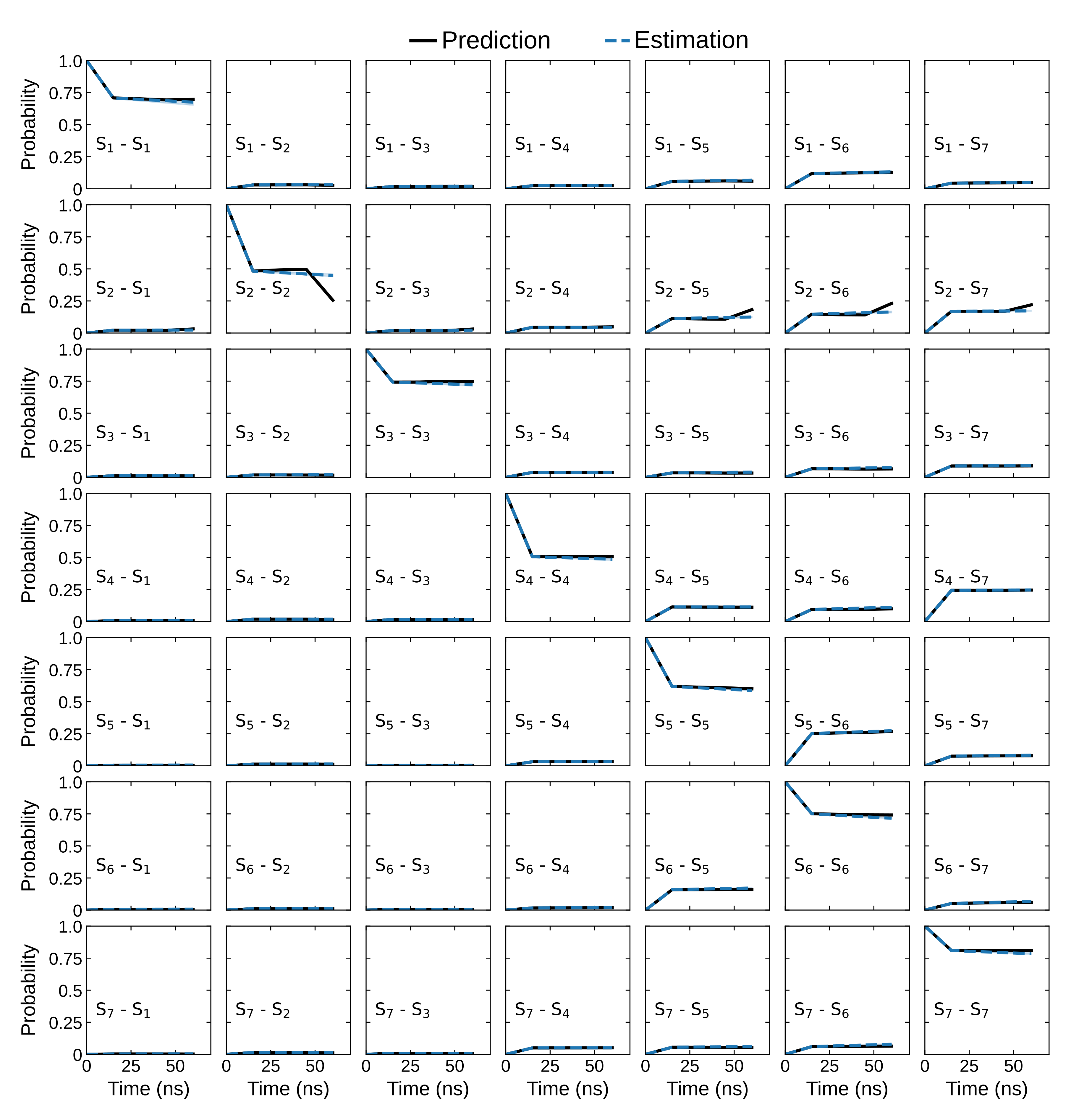

Supplement: S10 Fig — (TIFF) [file pcbi.1010583.s010.tiff]

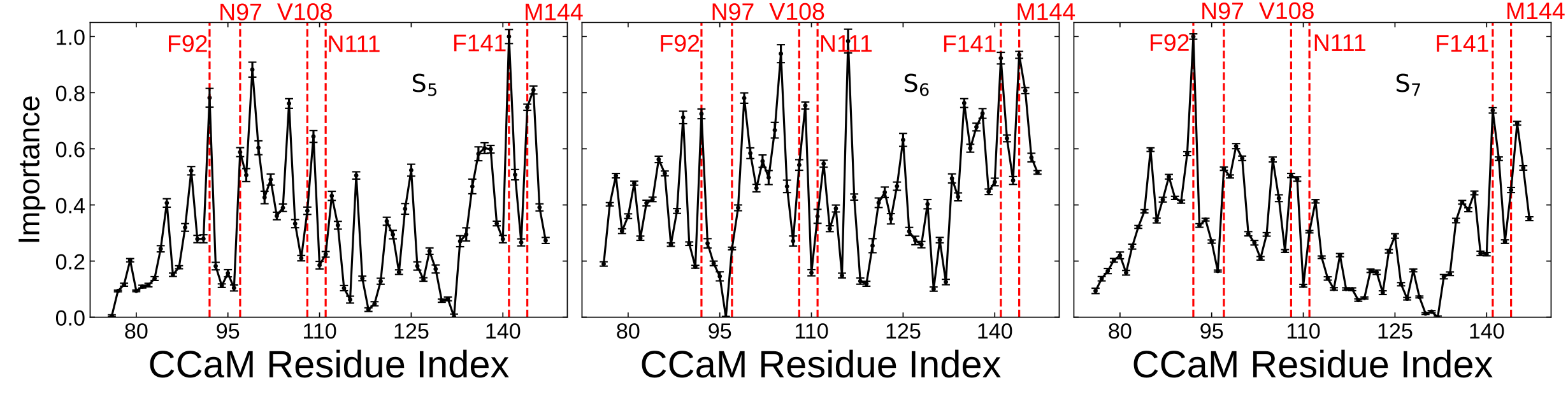

Supplement: S11 Fig — Plots represent the mean values calculated from five-fold cross-validation and the standard deviations are plotted as error bars. Physiologically important residues and those with high importance values are illustrated using red dotted lines. (TIFF) [file pcbi.1010583.s011.tiff]

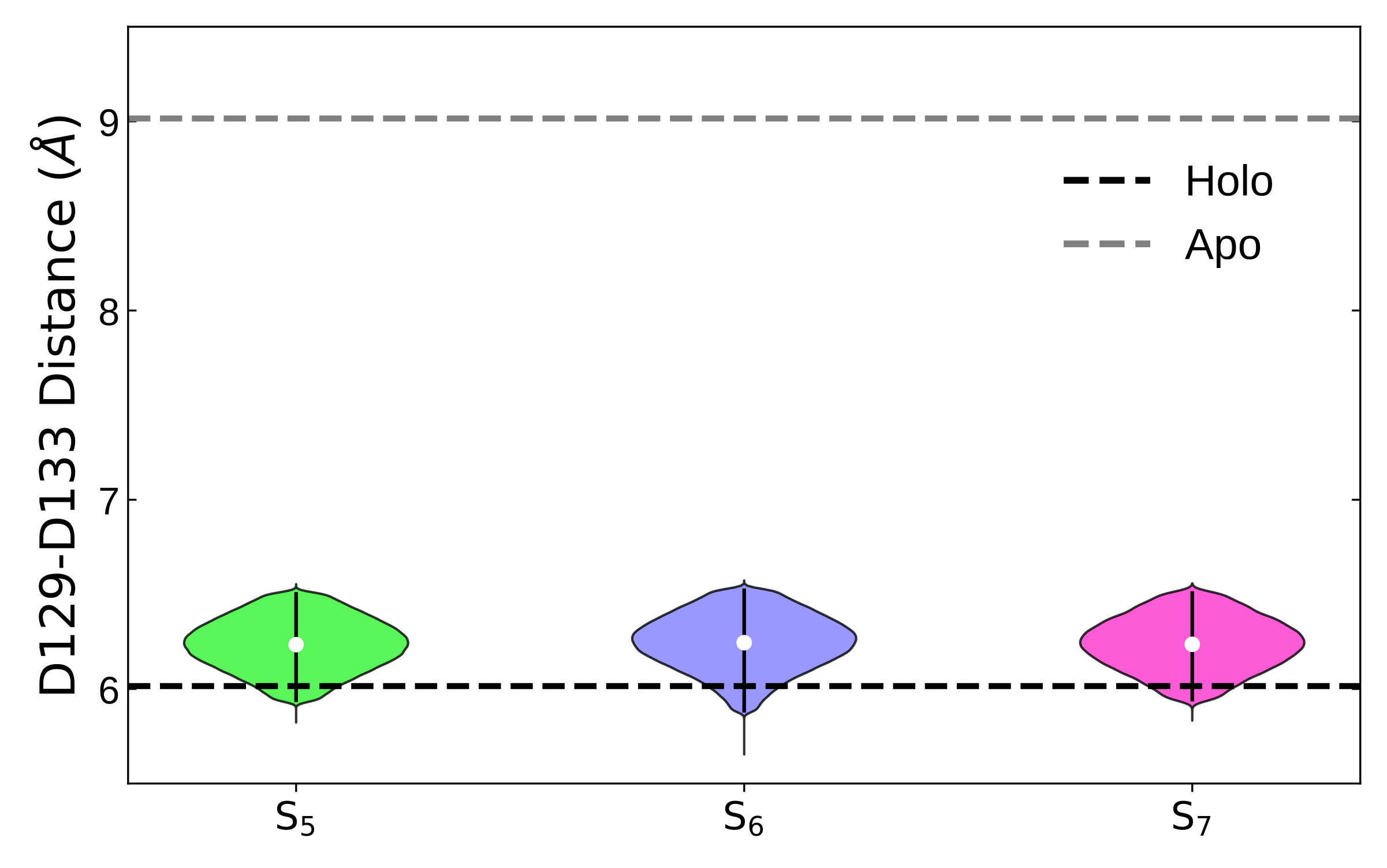

Supplement: S12 Fig — Each violinplot spans the 5th and 95th percentile of distances and is weighted by the Markov state model probabilities. The median value for each macrostate is represented as a white dot. The inter-residue distances calculated from apo- (PDB: 1CFD) and holo- (PDB: 1CLL) calmodulin structures are shown as grey and black dotted lines respectively. (TIFF) [file pcbi.1010583.s012.tiff]

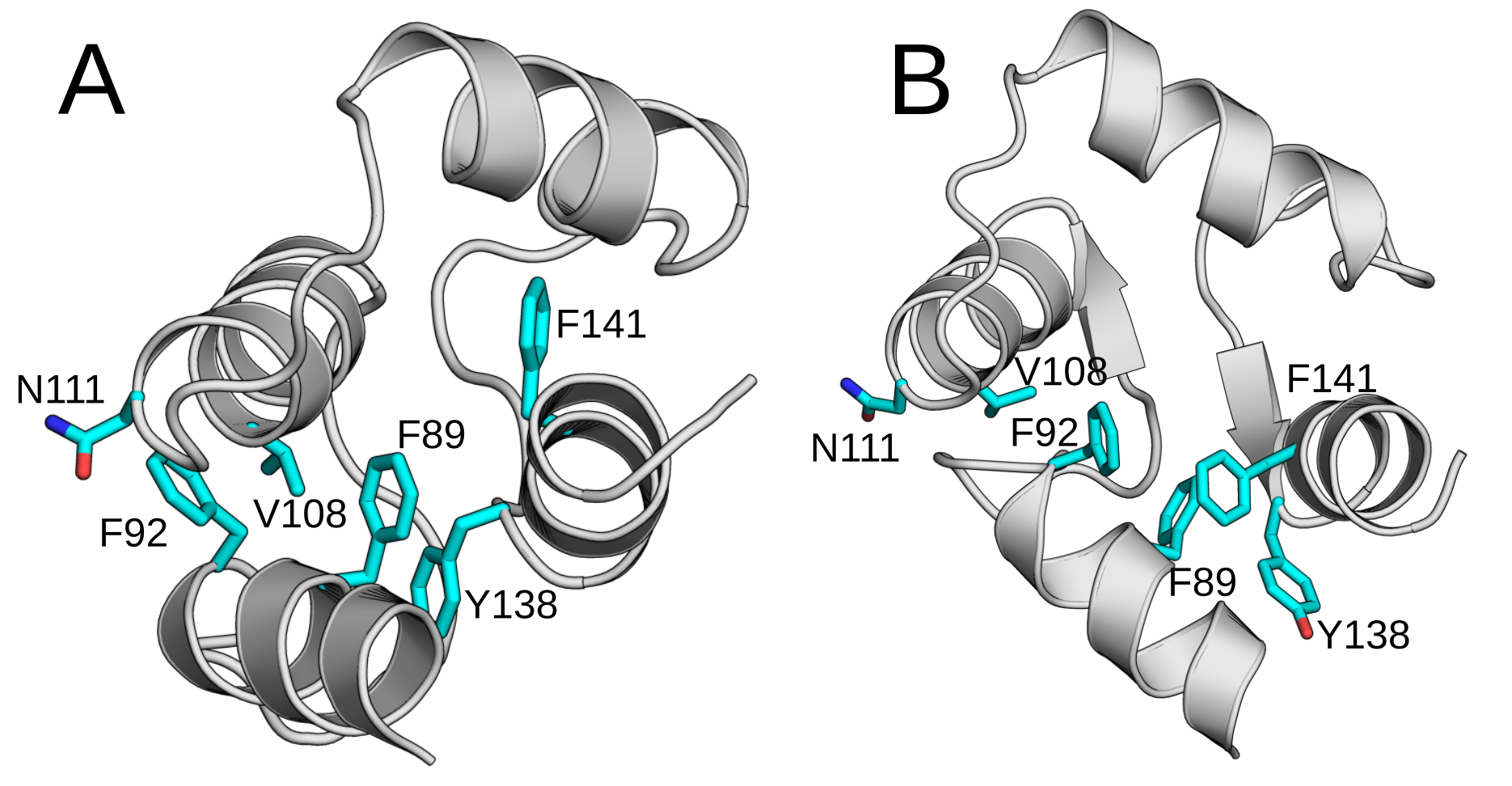

Supplement: S13 Fig — Transition in the stacking of aromatic residues between the (A) apo- (PDB: 1CFD) and (B) holo- (PDB: 1CLL) states of calmodulin induced by the binding of Ca2+ ions. (TIFF) [file pcbi.1010583.s013.tiff]

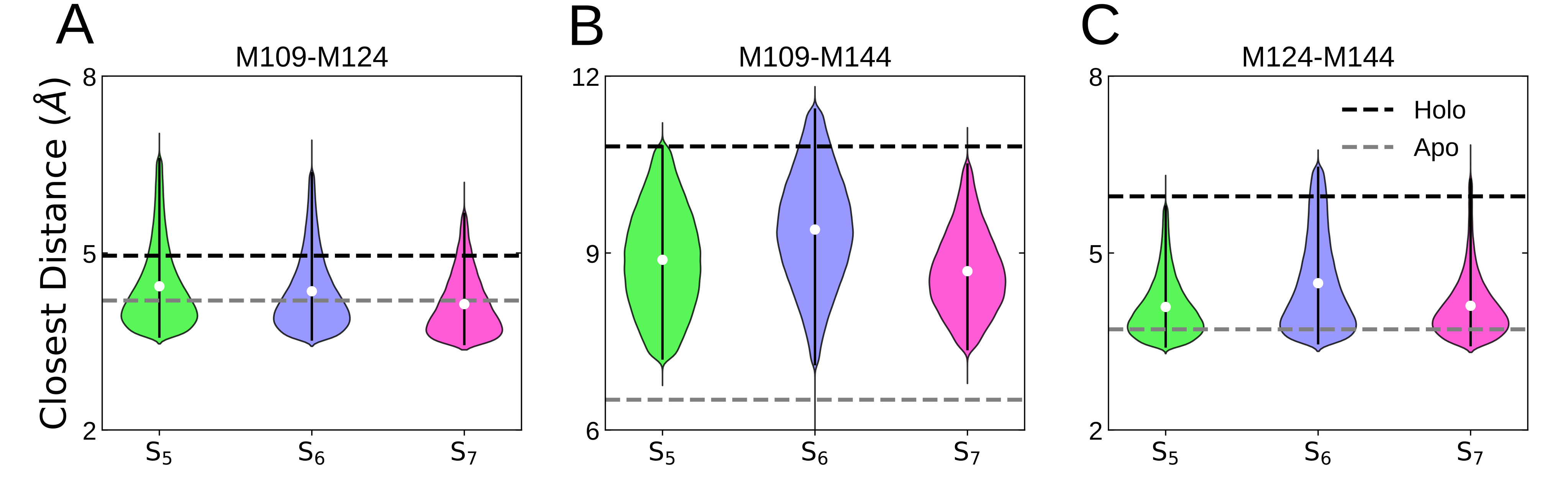

Supplement: S14 Fig — Each violinplot spans the 5th and 95th percentile of distances and is weighted by the Markov state model probabilities. The median value for each macrostate is represented as a white dot. The inter-residue distances calculated from apo- (PDB: 1CFD) and holo- (PDB: 1CLL) calmodulin structures are shown as grey and black dotted lines respectively. (TIFF) [file pcbi.1010583.s014.tiff]
