# Supplementary material for: Markov state modelling reveals heterogeneous drug-inhibition mechanism of Calmodulin
Source: PLoS Comput Biol. 2022 Oct 7;18(10):e1010583. doi: 10.1371/journal.pcbi.1010583 (PMC9581412; doi:10.1371/journal.pcbi.1010583)
Supplement: Attachment — Submitted filename: Reviewer_comments.pdf [file pcbi.1010583.s015.pdf]

We thank the reviewers for the useful comments that have helped us improve the manuscript. Responses to the suggestions are given inline here in ***Bold*** *and the associated changes are highlighted within the manuscript.*

Reviewer #1

The authors combined molecular dynamics simulations, markov state model and machine learning strategies to investigate the structural dynamics of CaM exerted by the binding of one known drug TFP, which prevents the downstream protein associations. The authors claimed that the TFP binding to CaM can put an influence on its C-terminal region and induce it into a Ca^{2+}-free conformation. This current manuscript provides some structural insights into the regulatory roles of TFP in stabilizing CaM. Despite that, many technical flaws should be fixed and more structural analyses should be provided in the revised version before the final acceptance. See below:

We thank the reviewer for the comments and suggestions. Please see our point-by-point response below.

1. To validate the MSM, I would suggest the authors to perform the GMRQ analyses for the hyperparameters selections of the MSM, including the tICA lag-time, microstate number, number of tICs etc.

Following the reviewer's suggestion, we utilized the generalized matrix rayleigh quotient (GMRQ) to validate the choice of MSM parameters. For each of the choice of ligand featurization (5 Å, 5Å _inv, 6 Å, 6Å _inv, 7 Å, 7Å _inv, 8 Å, 8Å _inv), we calculated GMRQ score across number of tICs and k-means microstates (Figure S4 in the revision).

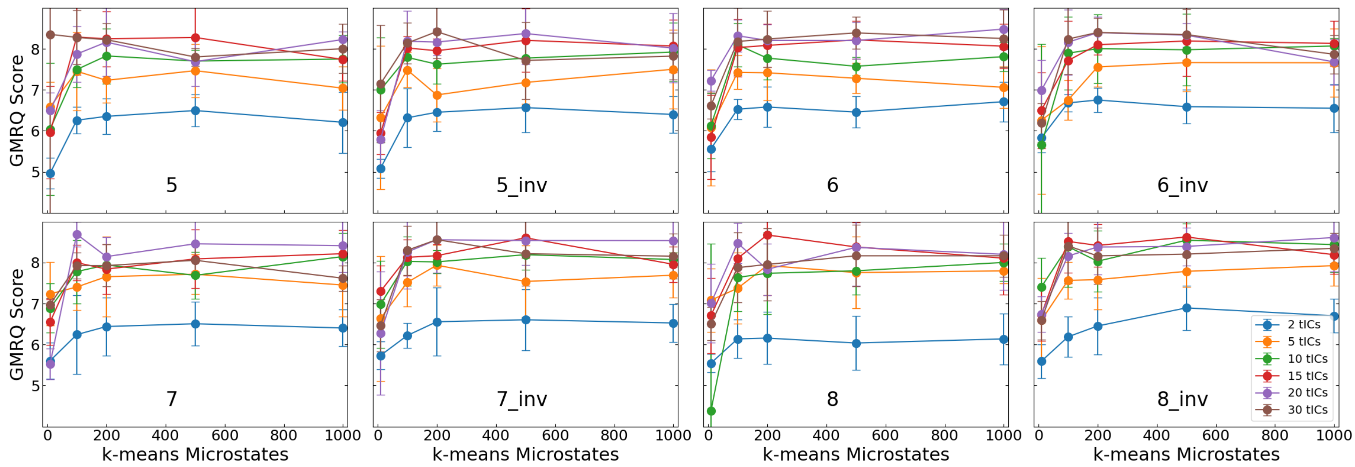

First, the results validate the VAMP2 calculations showing that choice of featurization method does not impact the MSM substantially. Additionally, in each case, we see no improvement in GMRQ score with increasing the number of tICS & microstates beyond the 10 tICs and 200 microstates we had initially reported. In addition to inserting a supplementary figure, we have edited the text to reflect the inclusion of this additional analyses for hyperparameter selection.

Related to this, the implied timescale curves shown in Fig. S2 are actually not well levelled off, I suggest the authors to further increase the lag-time to check if the curves can converge or try alternative models.

The reviewer makes a good point: the implied timescales could theoretically level off even more and the aim should be to pick a lag-time after levelling off of the implied timescales. Nevertheless, obtaining such a behaviour for complex processes of biological significances is known to be challenging and choices similar to ours are routinely published (see for example Fig. S6 in

https://pubs.acs.org/doi/abs/10.1021/acs.jpcb.9b08257, Fig. S6 in https://doi.org/10.1016/j.bpj.2022.01.024, Fig. 2 suppl. 2 in https://elifesciences.org/articles/68369#fig2s2, Fig. S8 in https://doi.org/10.1038/ncomms11244, amongst many others). Indeed, while a choice of increase in lag-time would in theory improve the Markovianity of the model, it would do so at the detriment of reducing the data available to build the transition matrices. We thus stand by our choice of lag time, as it indeed yields a Markovian model, as evidenced by the results of the Chapman-Kolmogorov test reported in Figure S9. Please refer to the answer to reviewer 3's point 6 for a more extensive discussion of the convergence of the MSM using this lagtime.

2. In Fig. 2 and Fig. 3, the errors of the stationary distribution and MFPTs are not provided, this should be fixed in the revised manuscript.

We have edited Fig. 2 and Fig. 3 to include the calculated standard deviations of the MFPTs and stationary distributions.

3. From Fig. 1D, TFP can potentially bind with CaM in different pockets and varied TFP/CaM ratios. The authors should provide more details regarding the system setups. For example, how many TFP molecules were included in the system? How to parametrize the TFP molecule in the study etc. One structural model for the complete system is recommended.

In this work, we have only considered the binding pose and mechanism of a single TFP ligand binding at the intra-lobe C-CaM site. The simulation system thus consisted of the whole CaM molecule (N-CaM & C-CaM), bound Ca^{2+} ions and 1 TFP molecule located within C-CaM. The Data Availability section of the manuscript refers to the Zenodo repository (10.5281/zenodo.6626415) where a pdb file of the modelled system is available.

The Charmm compatible TFP parameters were generated using STaGE and we have updated the above-mentioned Zenodo repository to contain the Gromacs .itp file describing the TFP parameterization.

We have edited the methods section to include these details. In addition, to further improve clarity, we have added SI Figure 1 that visualizes the C-CaM states 2, 5 & 6 from our prior work that made up the simulation seeds.

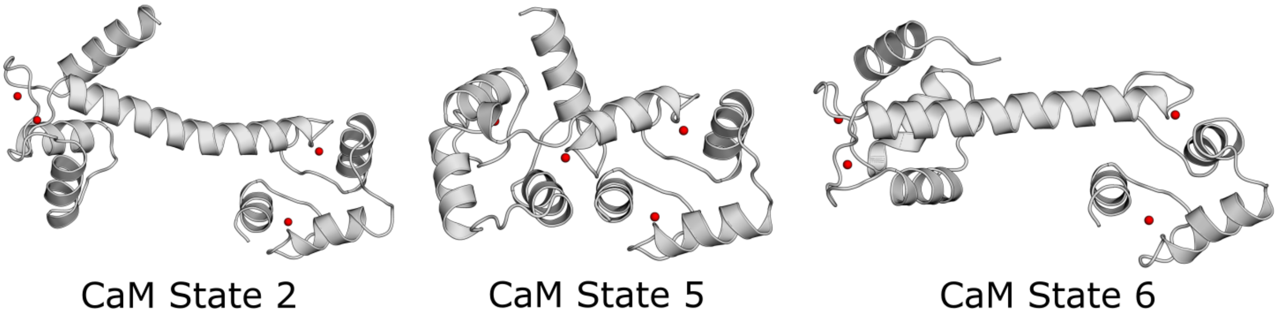

4. In Fig. 4, the authors highlighted several critical residues in CaM, however, no detailed structural analyses to support their conclusions. I would suggest the authors to conduct more structural analyses and discuss with their ML results.

To identify the molecular basis for the calculated importance of critical residues, we added a new Fig. 4B that calculates the changes in inter-residue distances between the three S_5, S_6 and S_7 states. Consistent with the calculated importance, the F92 and F141 residues displayed changes in inter-residue distance.

Additionally, the relative inter-residue distance calculations suggested the basis for the predicted V108 and N111 residue importance was their ability to intercede the aromatic stacking. We have edited SI Fig. 12 to include the positioning of these residues in the holo- and apo-CaM states.

5. The authors attempted to compare the TFP-bound CaM with its Ca2+-bound/unbound state, however, neither Ca2+-bound or -unbound CaM has been simulated in current work. I would suggest the authors to perform several additional MD simulations for these systems in order to make a valid comparison.

We thank the reviewer for the suggestion. While no simulations to study the impact of retaining/removing the Ca^{2+} ions were performed here, in our previous work (Westerlund and Delemotte, PLoS Comp. Biol., 2018), we have extensively modelled the impact of the ions on CaM conformational states. We have re-analyzed the data in the current context and edited Fig. 4 & 5 to additionally include the mean and standard deviations of distance distributions within the prior MD simulations. The results support the hypothesis within the manuscript where opposite TFP orientations can trigger an apo-like Calmodulin transition.

Reviewer #2

Overall, the authors detail the various mechanisms of TFP interactions with Calmodulin (CaM) by performing molecular dynamics simulations and adaptively sampling the conformational landscape of the CaM-TFP complex. The authors aim to explain the mechanism of TFP inhibition, and use extensive molecular dynamics simulations for the same. The authors use a guided machine-learning and quantitative approaches to explain the binding modes of TFP to CaM, which gives further validity to the results. They aim to explain the hydrophobic rearrangements of the binding pocket are responsible for the inhibition of CaM. It is an interesting work and I have a long list of minor edits. Further review is not needed.

We thank the reviewer for the appreciative comments and noticing the oversights within the manuscript.

Introduction:
Paragraph 4: Inconsistent terminology is used – intra-lobe is referred to as within-lobe and vice-versa.
Paragraph 5: 'MD Simulations may be used?' Authors could change this line to "MD simulations are routinely used"

We have used the suggested phrasing within the manuscript.

Methods:

Calmodulin-TFP system preparation and equilibration: Was the N-Terminal Domain present in the simulations, but the TFP only bound to the C-Terminal domain? The manuscript does not make this clear. The authors say 'TFP was placed in prominent states' – can they make the said 'placement' clear? What are states 2, 5 and 6 from prior work? Can the authors justify their choice? From how many total states were these three chosen? Were these simulations in the presence of calcium ions? Please make the methodology more clear.

*The simulation system consisted of the whole CaM molecule (N-CaM & C-CaM), bound Ca^{2+} ions and 1 TFP molecule within C-CaM. The Data Availability section of the manuscript refers to the Zenodo repository (*10.5281/zenodo.6626415*) where a .pdb file of the modelled system is available.*

In our prior work (Westerlund & Delemotte, PLoS Comp. Biol., 2018), we sorted C-CaM into configurations suited to bind partner proteins in a shallow, intermediate, and deep manner. The three 2,5 and 6 C-CaM states used to seed simulations here are clustered configurations from REST simulations that best describe each of these states respectively. The initial TFP configuration was generated by alignment of the C-CaM Ca atoms of each of the three states to chain A of the 4RJD crystal structure. Within state 2 – to compensate for reduced ligand diffusivity due to the collapsed hydrophobic core, we initiated additional REST simulations with ligand configuration generated by aligning to chain B of the 4RJD structure.

We have expanded the methods section within the manuscript and additionally added SI Figure 1 to visualize the C-CaM states 2, 5 & 6 from our prior work that made up the simulation seeds.

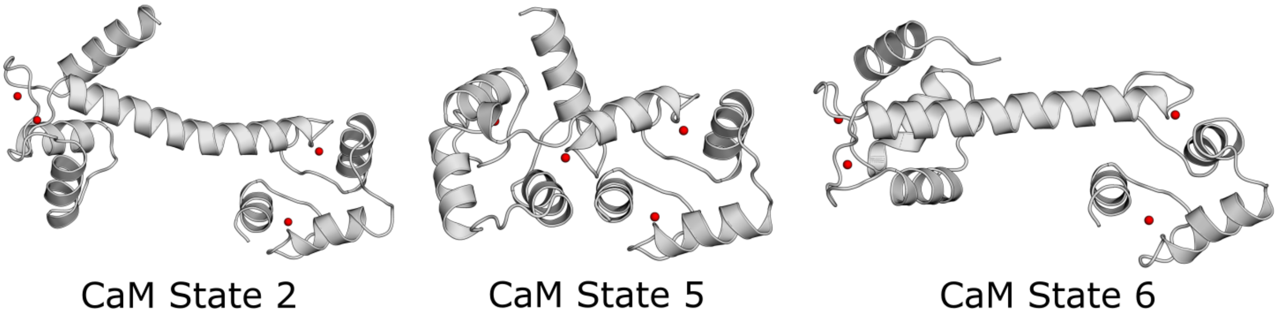

Replica Exchange solute tempering and adaptive sampling simulations: The authors perform Replica Exchange Simulated Tempering (REST) simulations. However, there is not enough motivation provided for the rationale behind these simulations. Why are the authors performing these simulations? A mention of the method in the introduction with some relevant background is needed.

The REST simulations were only utilized to obtain a sufficiently wide breadth of C-CaM and TFP states to serve as seeds for the initial round of adaptive sampling. The REST simulations were NOT used for analysis or MSM construction. Within the Methods section of the revised manuscript, we have added a sentence discussing the motivation for performing the REST simulations. We have also discussed the analysis of the REST simulations' efficiency and added a SI Figure 2 validating the Replica overlap and exchange rates.

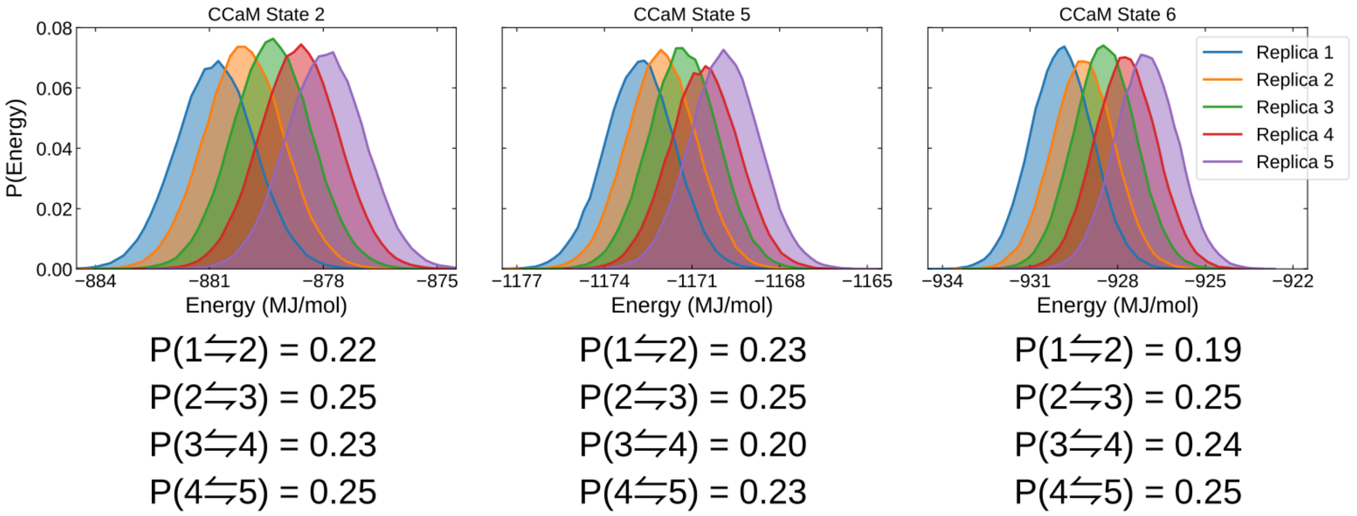

The conformations were chosen uniformly over the grid – what was the criteria for ensuring uniformity? Could the authors provide more details into the data collected per round of sampling?

We have expanded the methodology section within the revised manuscript to provide more information on the adaptive sampling used. In the work, to initiate the first round of sampling of length 100 ns of each state, we projected protein-ligand contacts within the REST simulations onto two slowest tICs. For each CaM-TFP state, 10 points uniformly distributed across the two-dimensional grid spanning the extent of these two tICs were selected – and their representative conformations were used to initiate the first round of 100 ns long unbiased MD simulations. For each subsequent round of sampling, we built a MSM using the sampled trajectory frames and then selected microstates with a low stationary probability as seeds for subsequent simulation rounds.

Selecting distance-based features and projecting the data onto slow degrees of freedom "The minimum distances between all C-CaM residues and five TFP atoms distributed across the ligand's structure (C25, C24, C10, S, C16) were calculated." Why these 5 atoms in particular are chosen for TFP?

A subset of the atoms were chosen to expediently describe the ligand. The terminology 'distributed across' refers to the five chosen C25, C24, C10, SC4 and C16 atoms each being from the ligand's functional group (see Figure 1C). The C25 atom is within the aromatic ring and C10 is adjacent to the trifluoromethyl group. The SC4 Sulphur is situated between the two. Finally, the C16 and C24 atoms are situated within the alkyl linker and piperazine groups respectively. We have elaborated the phrasing within the Methods section to

'minimum distances between all C-CaM residues and five TFP atoms (C25, C24, C10, SC4, C16) encompassing the ligand's functional groups were calculated'

Is the S in (C25, C24, C10, S, C16) same as SC4 in Fig 1C? If yes, it is used inconsistently.

We thank the reviewer for identifying the inconsistency. 'S' and 'SC4' refer to the same Sulphur atom within Trifluoperazine. We have amended the text to refer the atom as SC4 throughout to be consistent with the notation in the shared PDB file on Zenodo.

Fig S1: What do the values in the legend 0.5, 0.6 indicate? Can the authors make the term 'feature type' clear?

In the revised manuscript, within the Methods section we have elaborated on the distance feature transformations attempted. We have expanded on figure caption for SI Fig. 1 to elaborate on the legend terminology. Additionally, to better correlate with the terminology in Å within the methodology section, we have changed the legend to 5, 5_inv, 6, 6_inv …

Construction of a Markov State Model: 'Results were stable' – can the authors make it more quantitative justification than a qualitative one?

To quantitatively validate the choice of MSM hyperparameters, we utilized the generalized matrix rayleigh quotient (GMRQ). For each of the choice of ligand featurization (5 Å, 5Å _inv, 6 Å, 6Å _inv, 7 Å, 7Å _inv, 8 Å, 8Å _inv), we calculated GMRQ score across a number of tICs and k-means microstates.

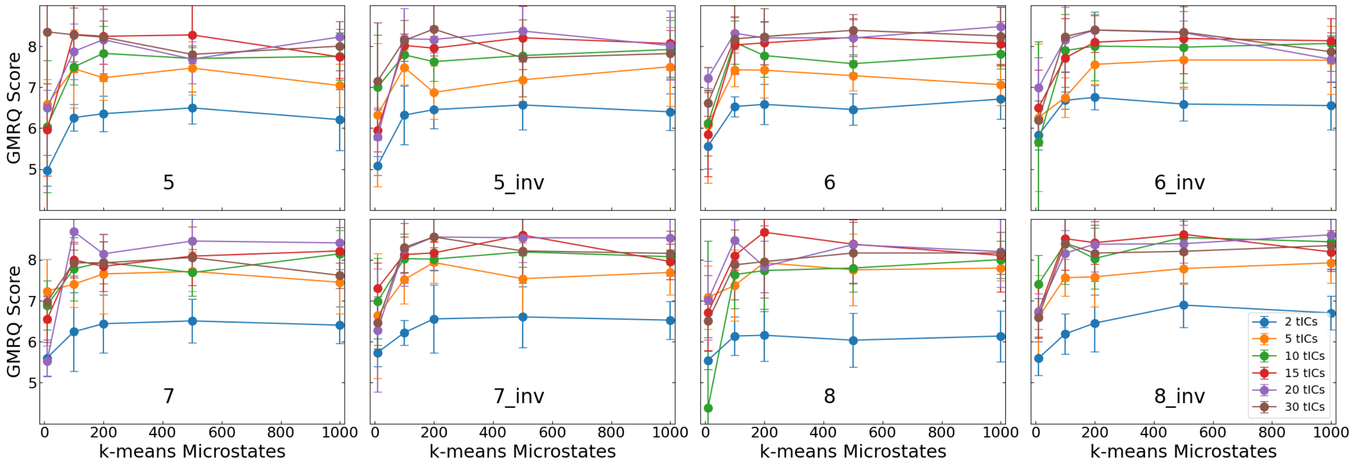

First, the results validate the VAMP2 calculations where the features equally well-described the system. Additionally, in each case, consistent with the choice of 10 tICs and 200 microstates for MSM construction, we see no improvement in GMRQ score with increasing the number of tICS & microstates beyond this. We have edited the text to reflect the inclusion of this additional analyses for hyperparameter selection.

Fig S2: Have the authors computed the errors for the implied timescales?

The errors for the implied timescales were calculated using Bayesian inference. The shaded regions of the plot represent the 95% confidence intervals for the calculated implied timescales. We have expanded the captions of SI Fig. 5 to better explain this.

Identification of important residues using machine learning: The cutoffs for the MSM construction (6Å) differ from the cutoff used for identification (6.5Å) – can the authors justify this decision?

The feature types used for MSM construction and machine-learning residue identification are different. MSM construction considered distances between TFP atoms and C-CaM residues. In contrast, the machine learning algorithms utilized inter-residue distances as features. However, to validate the results, we recalculated the state-dependent RF importance using a 6 Å cutoff and found no noticeable difference in the residues identified.

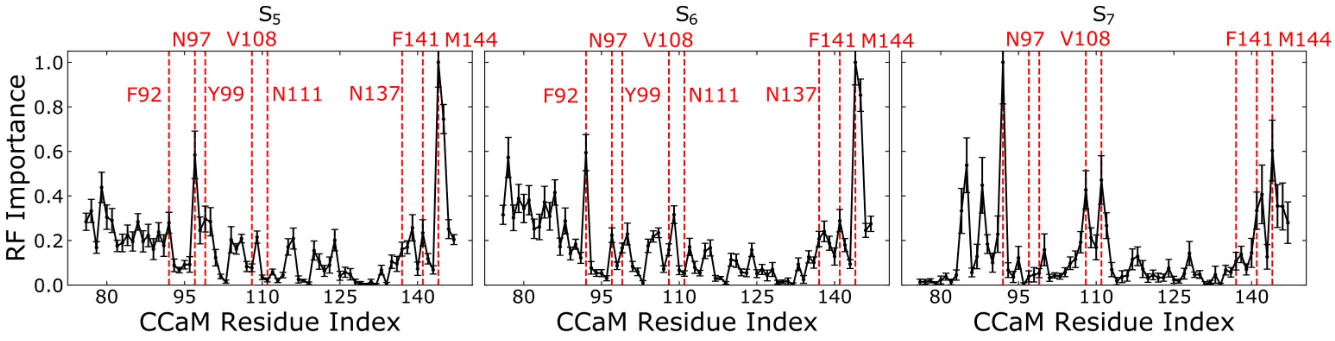

Results and discussion:

Validation of Markov state model: The Chapman-Kolmogorov state test differs for the S2-S2 conversion diverges significantly between the predicted and the estimated probabilities.

The reviewer is right to note the S2-S2 conversion diverges within the Chapman-Kolmogorov test. However, the state makes-up an insignificant 2% of the equilibrium distribution. The more populated S_5, S_6 and S_7 states display well-validated Chapman-Kolmogorov tests.

Why tIC1 and tIC3 were chosen as the slowest metrics? Why not tIC2?

The projection of the free-energy surfaces along tIC1 and tIC3 was chosen only for visualization purposes as it offered a crisp separation of states when plotted in 2D space (SI Fig. 7). For the actual purposes of k-means clustering and MSM construction, we used the 10 slowest processes.

The metastable states represent different binding modes: The slowest process corresponding to tIC1 – shows significant separation among macrostates S5 and S6 in Fig 2B, but these two macrostates represent similar binding modes. Why?

The 10 slowest tICs were utilized for k-means means clustering and MSM construction. While the S5 and S6 states with similar binding modes are separated along tIC1, the states display separation from S7 along most of the other 9 slow processes. We have added this projection onto the 10 tICs as SI Fig. 8 within the revised manuscript.

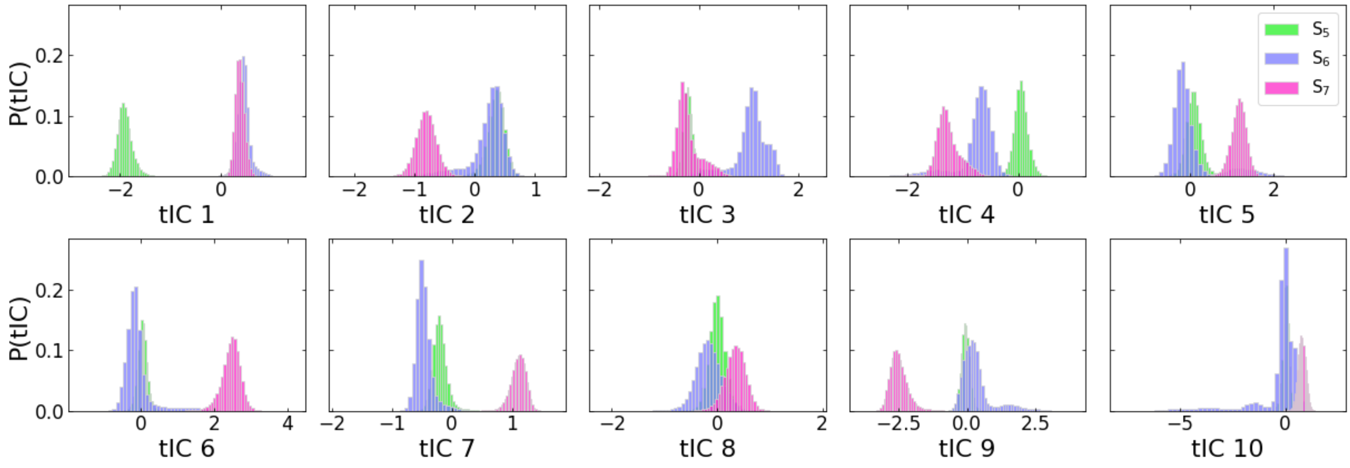

Authors mention an orange circle and cyan circle, in Fig 3 but it seems to be missing.

We have checked the figure and ensured that the circles are visible within the contact plots of Fig. 3B.

Is there evidence of the TFP interconverting through the different binding modes?

The SI Fig. 8 overlays the initial and final configurations of each individual trajectory onto the underlying free energy surface projected along tIC1 and tIC3. In multiple individual trajectories, we observed transitions into/out of basins (binding modes). In fact, in multiple trajectories, we observed direct transition between the basins – highlighted in magenta within the Figure.

TFP Binding modes alter the C-CaM binding pocket by affecting local interactions at a Ca+2 binding site The C_α RMSD of 1.71Å is unclear – is it between S5-S6, S6-S7 or S5-S7?

We calculated the C-CaM Cα RMSD between the representative structures of all three (S5, S6, S7) states and 1.71 Å is the maximum divergence. Specifically, this is between the S5 and S7 states. We have added a clarification for this within the manuscript.

Can the authors give a more qualitative explanation of the y-axis in Fig 4A – what does RF importance signify? How does RF importance correlate to the dynamics of the protein macrostates S(5-7)?

A high RF importance indicates large state-dependent changes in the residue's interaction pattern and thereby suggests that the residue moves with respect to its interaction partners. To rationalize the structural basis of the calculated RF importance, we added Fig. 4B that calculates the change in mean inter-residue distances between the states. The new plots suggest the importance of the V108 and N111 within S_7 to stem from them lying along the same face of a helix whose motions impeded stacking within the C-CaM core (see Edited SI Fig. 12).

Flipping of the TFP molecule affects the hydrophobic pocket and is associated with a changed beta-sheet structure content. Have the authors performed simulations in the presence of Ca+2 to explore the reduced affinity binding between Ca+2 in the presence of TFP?

This is an intriguing scientific question to look at the inter-play between the effects of the inhibitor TFP binding and Ca2+ ions on Calmodulin. However, to be consistent with the current work, a similarly extensive repository of simulations of apo-CaM is necessitated. We thus concede that this is beyond the scope of the current manuscript.

Reviewer #3

In this manuscript, the authors have constructed Markov State Models (MSMs) from molecular dynamics (MD) simulations to study the binding of trifluoperazine to calmodulin. They identified important protein residues that are associated with several drug binding poses. Interestingly, they find that upon binding, trifluoperazine can stabilize both apo and holo-like calmodulin conformations, depending on the binding pose. Overall, this is a rigorous study, and the manuscript is also well-written. Their results provide new insights into the understanding of the calmodulin inhibition and may facilitate the drug development in the long term. Therefore, I would like to recommend its publications after minor revision (see my comments below):

We thank the reviewer for the appreciative comments.

1. To obtain the input features for the tICA analysis, the authors simply chose all the pair-wise distances between protein residues and five TFP atoms within a cut-off (6\AA selected by the VAMP2 score). I am wondering if the application of Spectral-OASIS, SRVs or other methods (see discussions in JACS Au, 1(9), 1330, (2021)) can help further refine the input feature set?

More interestingly, will these methods (Spectral-OASIS, SRVs, etc) can identify the same set of important residues (e.g., distance features between these important residues and TFP) as those obtained from their explainable AI algorithm after the MSM construction?

The reviewer is right, a slew of methods indeed exist for dimensionality reduction and feature selection. Amongst them, we have chosen to resort to two different methods for feature selection (VAMP + GMRQ) and to two different methods for assessing feature importance (KL Divergence and RF), reporting stable results across methods. A more extensive comparison indeed appears interesting but represents a considerable effort that would be better suited for a more methodologically-oriented paper. Here, we have indeed chosen to focus our insights into the atomistic details of a problem of biological and pharmacological interest and worry that including a more systematic comparison to other existing methods would dilute our message. We do note this reviewer's suggestion for our further work, however!

2. The current discussion of binding poses is very detailed in terms of the importance of protein residues but lacking in terms of their inhibitory effect. Specifically, it is not clear how the binding pose inducing the holo-like conformation contributes to the inhibition of calmodulin. I would like to suggest the authors to expand their discussions of the binding poses with respect to the subsequent inhibiting effect to benefit a general audience.

To rationalize the structural basis of the calculated RF importance, we added Fig. 4B that calculates the change in mean inter-residue distances between the states. Consistent with the predictions, the distances between the helices comprising F92 and F142 residues are further apart in S_7 than in S_6/S_5. Additionally, the result suggest the importance of the V108 and N111 within S_7 to stem from them lying along the same face of an helix whose motions impeded stacking within the C-CaM core (see Edited SI Fig. 12). We have expanded the discussion of our results to reflect the above.

3. It will be helpful to include a SI figure to display the efficiency and convergence of their REST simulations (e.g., acceptance probability, coverage of the replica space, etc).

The REST simulations were only utilized to obtain a sufficiently wide breadth of C-CaM and TFP states to serve as seeds for the initial round of adaptive sampling. The REST simulations were therefore not used for analysis or MSM construction. Within the Methods section of the revised manuscript, we have added a sentence discussing the motivation for performing the REST simulations.

Complete convergence of the REST simulations is thus extraneous considering the multiple rounds of adaptive sampling. However, to validate the efficiency of the REST simulations, we added the overlap between adjacent replicas and acceptance probability to SI Fig. 2.

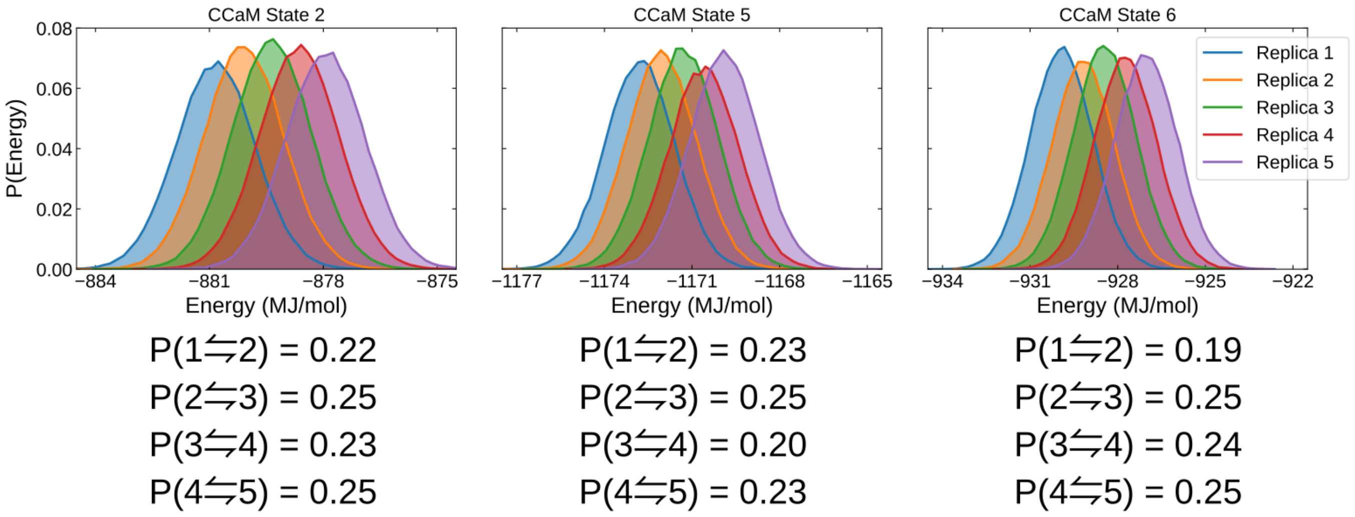

4. Fig S1: it is not obvious to me that "the feature type given by a 6 \AA cutoff and inverse distances yielded the highest mean score across lag-times". Could the authors clarify on this point?

The reviewer is right to note that VAMP2 calculations suggest the featurization methods to perform similarly in describing the ligand interaction. SI Fig. 3 was amended to include the calculated mean values across lag-times and the text was revised accordingly.

5. The choice of hyperparameters, including tICA lag time, number of tICs, and number of microstates can all impact the quality of an MSM. These hyperparameters can also be optimized using the cross-validation method based on the VAMP2 score or GMRQ. The authors may consider optimizing some of these parameters or include some discussions on this point. Especially on the choice of number of microstates, could the authors clarify on their criterion: "until the results described herein were stable"?

Following the reviewer's suggestion, we utilized the generalized matrix rayleigh quotient (GMRQ) to validate the choice of MSM parameters. For each of the choice of ligand featurization (5 Å, 5_inv, 6 Å, 6_inv, 7 Å, 7_inv, 8 Å, 8_inv), we calculated GMRQ score across number of tICs and k-means microstates.

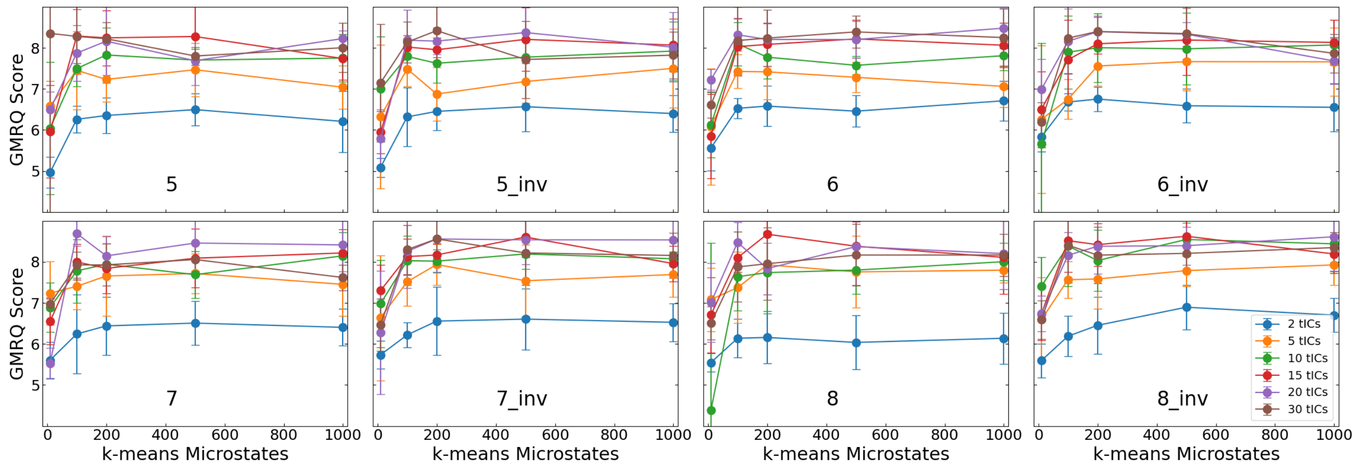

Consistent with the choice of 10 tICs and 200 microstates for MSM construction, we see no improvement in GMRQ score with increasing the number of tICS & microstates beyond this. We have added the GMRQ calculation as SI Fig. 4 and amended the discussion on the choice of tICs and microstates.

6. Fig. S2: it's not obvious to me that the implied timescales are fully converged. These implied timescale plots still display noticeable deviations from being flat even on the logarithm scale. Can the authors rephrase their claim of convergence and explain their choice of a relatively short MSM lag-time of 15ns? I notice that their 7-macrostate MSM (Fig. S6) is well validated by the Chapman-Kolmogorov test. Maybe adding the Chapman-Kolmogorov test of the 200-microstate MSM (on the residence probabilities of the top populated microstates) can help justify their choice of the Markovian lag time?

The reviewer makes a good point: the implied timescales could theoretically level off even more and the aim should be to pick a lag-time after levelling-off of the implied timescales. Nevertheless, obtaining such a behaviour for complex processes of biological significances is known to be challenging and choices similar to ours are routinely published (see for example Fig. S6 in https://pubs.acs.org/doi/abs/10.1021/acs.jpcb.9b08257, Fig. S6 in https://doi.org/10.1016/j.bpj.2022.01.024, Fig. 2 suppl. 2 in https://elifesciences.org/articles/68369#fig2s2, Fig. S8 in https://doi.org/10.1038/ncomms11244, amongst many others). Indeed, while a choice of increase in lag-time would in theory improve the Markovianity of the model, it would do so at the detriment of reducing the data available to build the transition matrices. We have revised the text to reflect the above.

Building on the reviewer's suggestion, we first calculated that at the chosen lag-time of 15 ns, we could resolve 34 processes.

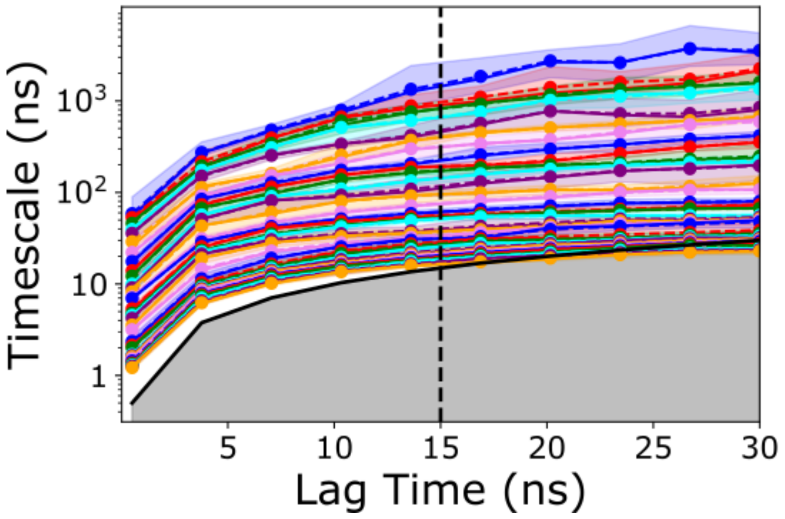

Figure 1: 34 eigenvalues of the transition probability matrix with timescales larger than the chosen lag-time of 15 ns (dotted line).

Following this, the Markov-model constructed with a lag-time of 15 ns appears well-validated by a Chapman-Kolmogorov test of the 35 microstates.

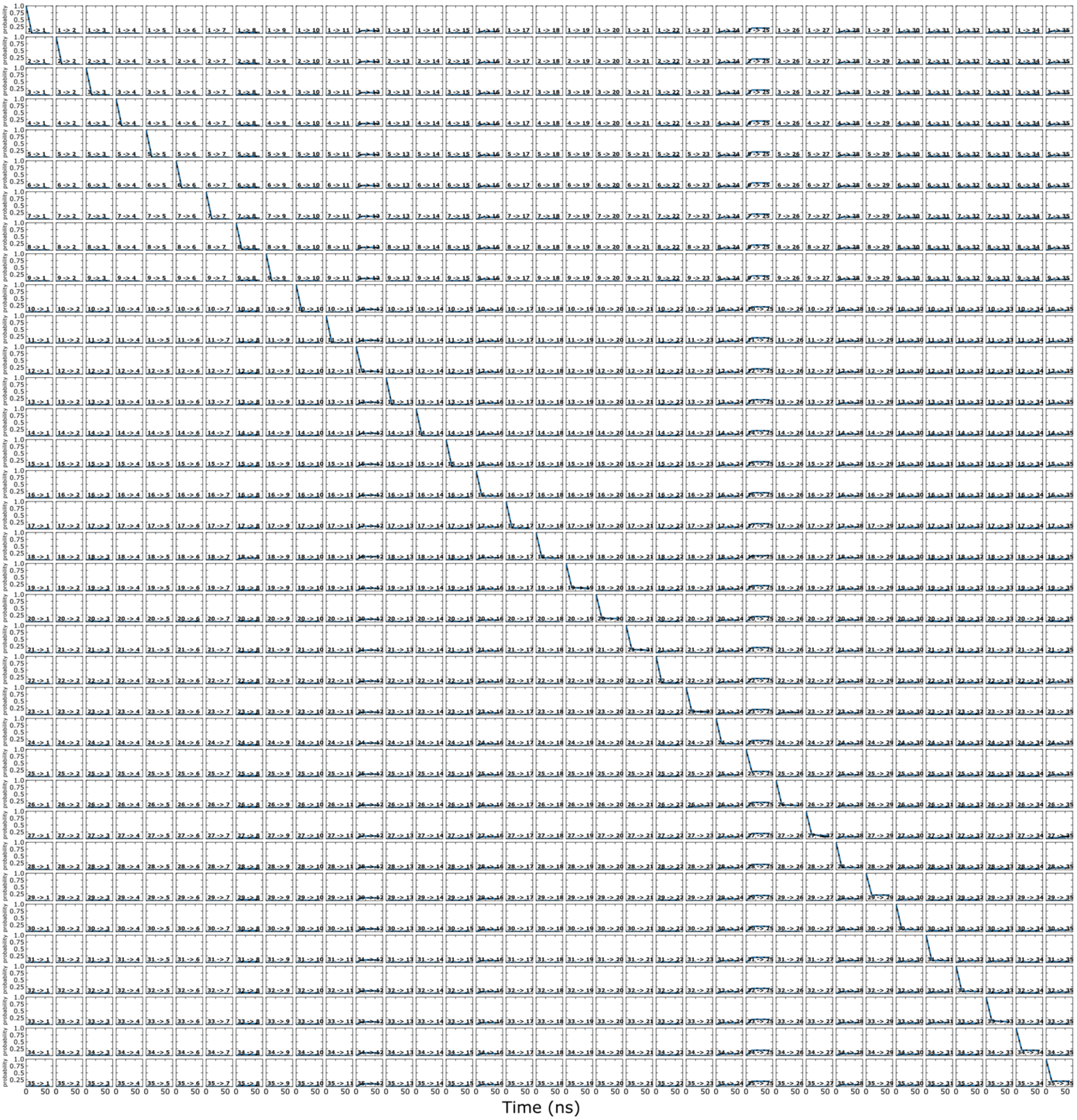

Figure 2: Markov state model validated by comparing the probabilities of transiting between the 35 microstates (blue line) and the calculated probabilities from the constructed model (black line).
